# Tuning of Thermopower in Molecular Junctions by Molecularly Controlled Sculpting of the Density of States in Their Leads

**DOI:** 10.1021/acs.nanolett.6c02296

**Published:** 2026-07-20

**Authors:** Mor Cohen Jungerman, Shachar Shmueli, Pini Shekhter, Yoram Selzer

**Affiliations:** † Department of Chemical Physics, School of Chemistry, 26745 Tel Aviv University, Tel Aviv 69978, Israel; ‡ The Tel Aviv Center for Nanoscience and Nanotechnology, Tel Aviv 69978, Israel

**Keywords:** Thermopower, molecular junctions, built-in potential, quantized states, semimetal, Fermi wavelength

## Abstract

Molecular junctions are promising for (low power) thermoelectric applications, providing their transmission landscape can be made sufficiently nonlinear at the Fermi level to efficiently break their electron/hole transport symmetry in response to a temperature gradient. We present a method to induce such nonlinearity leading to thermopower values of |*S*| > 0.5 mV/K. The method is applicable to metal–molecules–semimetal junctions in which a space charge region is formed within the semimetal with a characteristic length perpendicular to the interface that can be tuned to become comparable to the Fermi wavelength of the semimetal. Under such conditions, the interfacial density of states within this lead is quantized at energies that can be tuned to reside within ∼*k*_*B*_ *T* from the Fermi level by varying the molecular length. The resulting conductance behavior within this energy range becomes sufficiently steep to substantially break electron/hole transport symmetry, with ensuing high thermopower values.

## Graphical abstract

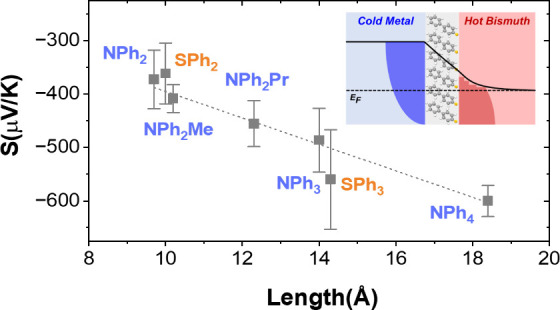

Thermoelectric (TE) devices provide renewable energy by direct thermal-to-electrical power conversion.^1,2,11–20,3,21–26,4–10^ Their efficiency is typically limited to a fraction of Carnot efficiency and is quantified by a dimensionless thermoelectric figure of merit: *zT* = *S* ^2^ *GT*/κ, where *T* is the absolute temperature, *S* is the Seebeck (thermopower) coefficient, and *G* and κ are the electrical and thermal conductance values, respectively. Most of the effort to improve *zT* wishes to maximize the power factor *S* ^2^ *G*.

A conceptional guideline for this endeavor can be formulated in the case of ballistic devices, using the Landauer formalism as an expression for *S*:^27^

1 $$S=-{\frac{\Delta V}{\Delta T}|}_{E=\varepsilon_{F}}=\frac{1}{eT}\frac{\int dE\tau \left(E\right)\left(-\frac{\partial f}{\partial E}\right)\left(E-\varepsilon_{F}\right)}{\int dE\tau \left(E\right)\left(-\frac{\partial f}{\partial E}\right)}≅\frac{\pi^{2}k_{B}^{2}T}{3e}\frac{1}{\tau \left(\varepsilon_{F}\right)}{\frac{\partial \tau }{\partial E}|}_{\varepsilon_{F}}$$

where *ε*_*F*_ and *f* are the Fermi energy and distribution respectively, τ is the transmission function, *k*_*B*_ is the Boltzmann constant, and *e* is the charge of an electron. Since in this equation the contributions of (*∂f*/*∂E*)­(*E* – *ε*_*F*_) above and below *ε*_*F*_ are identical in size and opposite in sign, high values of *S* necessitate a capability to manipulate τ­(*E*) to break this transport symmetry within the energy range defined by *∂f*/*∂E*.^13,14,16–22^ At low temperatures, this required control over τ­(*E*) is defined by the Mott formula on the right side of the above equation, as a requirement to tune a nonlinear conductance feature to the Fermi level of the system.

Several approaches have been considered for this purpose, including nanoscale structuring, band engineering and modulation doping, energy filtering, asymmetry in geometry, quantum confinement, meddling of topological properties, and formation of resonant states.^28,29,38–45,30–37^

In the case of molecular junctions operating as TE devices, the notion is that τ­(*E*) can readily be optimized for better performance by changing the structure of molecules.^46,47,56–58,48–55^ Yet, while based on the typical *G* and κ values of such junctions their Seebeck values need to be in the >0.5 mV/K regime to become relevant for applications,^26^ reported values of |*S*| in this range are rare and are often only a few tens of μV/K. The underlying reason for this is a lack of capability to control the process of energy-levels alignment within junctions, which takes place upon their formation and which too often results in “boring” τ­(*E*) landscapes near the Fermi level.

Two approaches have been suggested to overcome this problem. The first does not try to affect the process of energy levels alignment but instead devises junctions with pronounced steep nonlinear conductance regimes that span a broad energy range. For this purpose, molecules in which differences in the transmission phase of their conductance paths are engineered, with resulting destructive interference patterns in their coherent conductance properties. Junctions based on such molecules are indeed decorated by sharp features in their τ­(*E*) landscape close to the Fermi level.^46,47,56,57,48–55^ Yet, dephasing by various sources limits the efficiency of this approach to produce sufficiently high |*S*| values.^59^

The second approach wishes to affect the process of energy alignment by manipulating the character and density of states (DOS) of the leads. This can be achieved by covering them with adlayers of transition metal atoms, which are characterized by narrow d-bands.^60–62^ The renormalization process that the molecular levels undergo due to interaction with such bands, can result in substantial shifting of their energy toward *ε*_*F*_, and subsequently steeper conductance behavior at this energy. This process can be semiquantitatively analyzed by combining the Newns–Anderson model^63^ and the narrow d-band theory of Hammer–Nørskov that is commonly invoked to analyze catalysis by transition metal surfaces.^64^ While this approach is promising, it critically depends on the stability of the atomic adlayers, and the magnitude and resolution of the expected energy shift of the molecular levels are rather limited. Yet, this approach does demonstrate the potential of manipulating the DOS within leads as a viable tool to control and tune the transport properties of molecular junctions.^65^

Motivated by this reasoning and by relying on our previous work,^66–69^ we suggest here the following approach, which can be demonstrated by considering a general expression for transmission by tunneling, τ­(*E*) = $A\;\exp\left(-\frac{\sqrt{2m_{0}\Delta }}{\hbar }L\right)$ , where *A* is a constant, *m*_0_ is the mass of a free electron, ℏ is the Planck constant, and Δ and L are respectively the potential barrier and its length (defined here by the molecules).^76,77^ Using the Mott formula in eq 1 gives the following expression for *S*:

2 $$S=-\frac{\pi^{2}k_{B}^{2}T}{3e}\sqrt{\frac{m_{0}}{2\hbar^{2}}}\frac{L}{\sqrt{\Delta }}$$

According to the simple picture portrayed by eq 2, the effort to maximize *S* and the power factor (*S* ^2^ *G*) culminates mainly to minimize Δ. Using values typical for molecular junctions, such as Δ ≈ 1 eV and *L* ≈ 1 nm, results in *S* on the order of a few tens of μV/K. Additionally, since almost as a rule in molecular junctions their highest occupied molecular orbitals (HOMOs) are closer to the Fermi level than their lowest unoccupied orbitals (LUMOs), eq 1 suggests that, in the absence of a gate electrode, the Seebeck coefficient of molecular junctions is most likely positive.

The above expression for τ assumes a wide-band conditions, i.e., that *A* is energy independent. If, however, the DOS within the leads can be manipulated, this pre-exponential factor becomes energy dependent, *A*(*E*), and accordingly *S* changes to be

3 $$S=-\frac{\pi^{2}k_{B}^{2}T}{3e}\left[\sqrt{\frac{m_{0}}{2\hbar^{2}}}\frac{L}{\sqrt{\Delta }}+\frac{1}{A\left(E\right)}\frac{dA}{dE}\right]$$

The second expression in the brackets opens an additional route to maximize *S* and, as discussed below, also to optimize the power factor. The goal of the research described here is to demonstrate how *A*(*E*) that is basically the DOS within the leads can be molecularly manipulated and controlled to result in high Seebeck values.

Following the approach suggested in eq 3, we have recently presented molecular ensemble junctions (MEJs) with Seebeck values of >0.5 mV/K as a result of molecularly induced “spiky” features in the DOS of their leads located at energies within ∼*k*_*B*_ *T* from their Fermi levels (see Figure 1).^69^ The MEJs had a metal–molecules–semimetal structure in which a built-in potential can be formed within the semimetal, with magnitude that can be tuned by varying the molecular layers. Due to the relatively high dielectric constant of the semimetal and its small Fermi energy, the spatial extent of this built-in potential perpendicular to the interface can become comparable to the Fermi wavelength, and as a result, interfacial states become (2D) quantized and “spiky”. While our previous study^69^ indeed demonstrated junctions with high values of *S*, it still has some fundamental limitations that we wish to amend here. The first is the cumbersome capability it offers to precisely control the depth and shape of the confining potential wells within the semimetal and hence the energy values of the quantized states. This limited control results from the fact that the confining interfacial potential within the semimetal depends on three molecular attributes: the change imparted by the molecules to the work function of the semimetal, their length, and their dielectric constant (see quantitative discussion below). Here we show that by judiciously choosing sets of molecules, the number of free parameters decreases to one: the molecular length. The second limitation is the fact that, due to the large difference between the effective masses of (light) electrons and (heavy) holes, quantization is more easily formed for electrons as accumulation layers, i.e., below the Fermi level, and consequently with a resulting *S* > 0. Here we demonstrate that, by using conjugated molecules with sufficiently high dielectric constant, confinement within depletion layers is also possible and molecular junctions with *S* < 0 are accessible. The use of conjugated molecules is important also for the maximization of the power factor.

As in our previous studies,^66–69^ we demonstrate our approach using bismuth as the semimetal. Its weak electrostatic screening (relative permittivity *ε*_Bi_ = 100)^70,71^ and the small effective mass of its holes (*m*_⊥_ = 0.65*m*_0_ where *m*_0_ is the mass of a free electron)^72–74^ are both essential for the alteration of its DOS at the interface under confinement.

**Figure 1:**
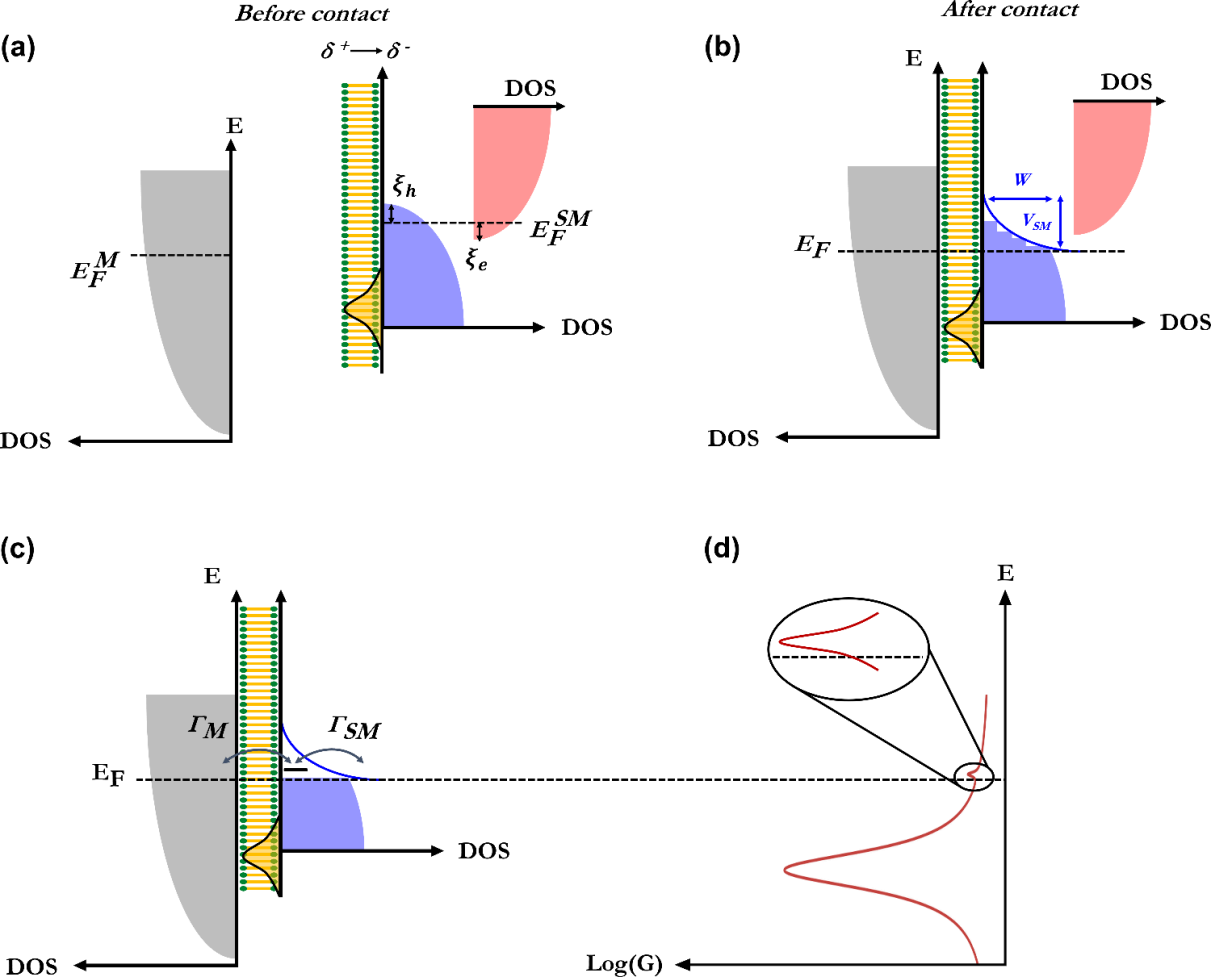
**a)** and **b)** The process of band alignment in metal–molecules–semimetal junctions. The DOS within the metal lead (gray) and the valence (blue) and conduction (red) bands of the semimetal are shown schematically along with their respective Fermi levels, *E*_*F*_^*M*^ and *ξ*_*h*_, *ξ*_*e*_. **a)** Before contact. As the adsorbed molecular layers in this study decrease the work function of the semimetal, its Fermi level, *E*_*F*_^*SM*^, is plotted at higher energy than the Fermi level of the metal. **b)** After contact, the Fermi levels are aligned, and a built-in potential, *V*_*SM*_, and a space-charge layer of width *W* are formed within the semimetal. As a result, the valence band at the interface is pushed up in energy and its DOS becomes quantized above the Fermi level within the potential well (see text for details). **c)** Focusing on a (2D) quantized level, its coupling (through the molecules) to the metal and to the bulk of the semimetal is defined by the coupling constants Γ_*M*_ and Γ_*sm*_, respectively. The conduction band, which is also shifted up in energy by the built-in potential, is not shown for clarity. The HOMO level responsible for the molecular transport is also shown (as an orange Lorenzian) to emphasize that the quantized state is formed near the Fermi level and deep within the HOMO–LUMO gap of the molecules. For clarity the LUMO is not drawn. **d)** The quantized state in the semimetal results in a spiky τ­(*E*) feature close to the Fermi level that is effectively responsible for the Seebeck magnitude and sign. Tuning of its energy is achieved by varying the length of the molecules, which in turn makes it possible to maximize the power factor.

A schematic presentation of the measurement setup is shown in Figure 2a. In all junctions, 300 nm thick Bi films were the bottom contact. As we have already shown,^66–69^ molecular monolayers with binding chemistry of thiols and amines can readily be assembled on Bi, and the formation process of such layers also strips off its native oxide. The monolayers of conjugated molecules used here (see Figure 2b) were formed by 3 h assembly from 1 mM (degassed) toluene solutions under inert atmosphere. Details concerning the chemistry of their binding to the surface, as well as data concerning their structural order, can be found in the Supporting Information (SI).

**Figure 2:**
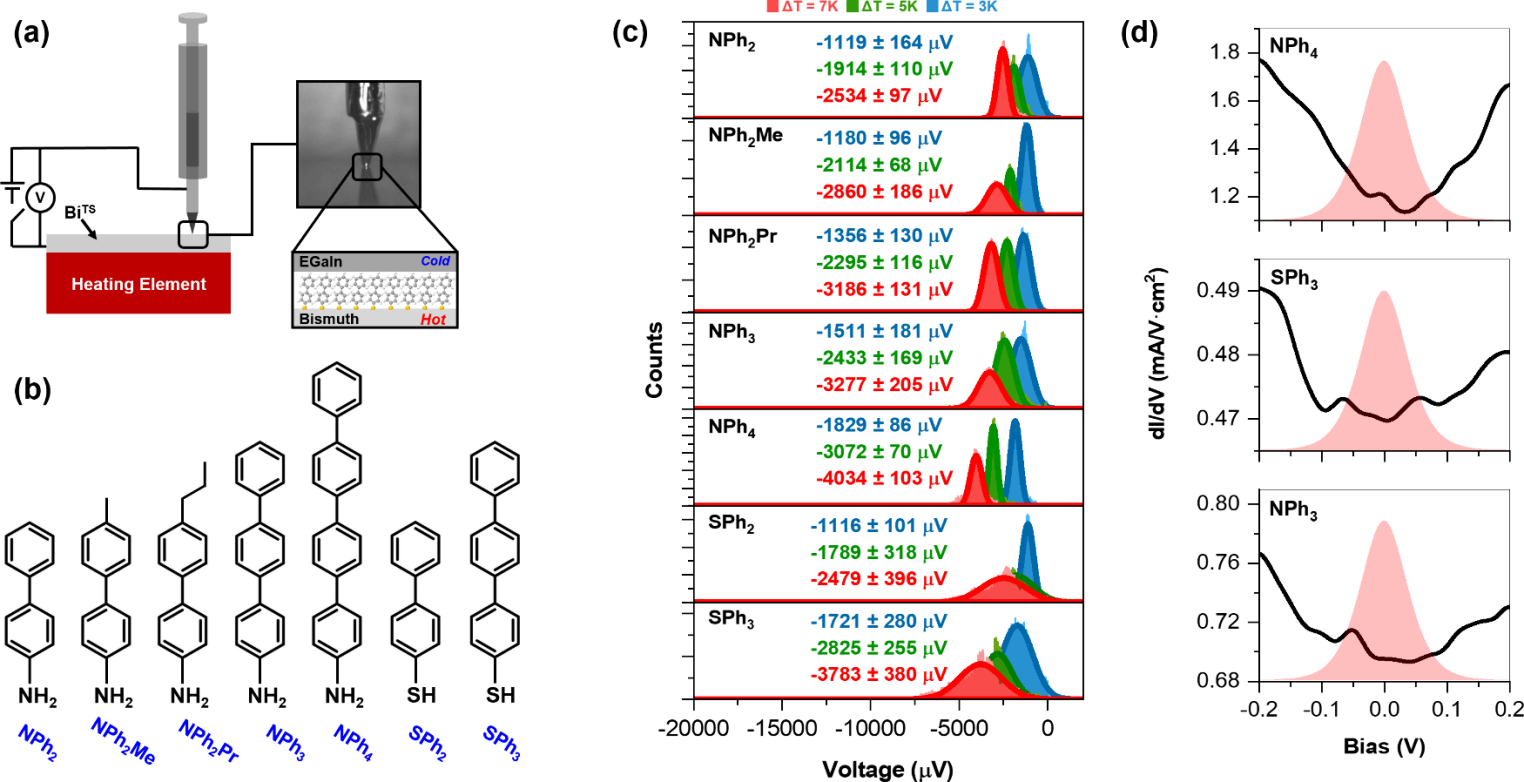
**The experimental setup and main results**. (a) A schematic presentation of an EGaIn/molecules/Bi junction with the basic circuit that enables both conductance and thermovoltage measurements. (b) The measured molecules and their abbreviations. (c) Histograms of the thermovoltage measurements for three different values of temperature gradients for each type of molecule. (d) Lock-in conductance measurements of junctions with the indicated molecules using a 20 mV ac signal at a frequency of a few tens of Hz. Assuming a symmetric potential divider, the observed levels according to these curves are located 30 ± 10 meV from *ε*_*F*_. Only junctions based on the longest molecules were sufficiently stable to survive these specific measurements. The energy window defined by *∂f*/*∂E* (see eq 1) is shown for each measurement as a pale red Gaussian.

A eutectic gallium–indium (EGaIn) drop was used as a top soft contact. All conductance and thermovoltage measurements were performed at room temperature under ambient conditions. The experimental setup and measurement procedures were identical to those described in our previous studies^66–69^ and very similar to those reported by others.^60,75–77^ Histograms of the measured thermovoltage of the different junctions under temperature gradients of Δ*T* = 3, 5, and 7 K are shown in Figure 2c. The data exhibits distinguishable mean values, indicative of the statistical significance of the results. The Seebeck values derived from these measurements are shown in Figure 3a. To the best of our knowledge these are the highest reported absolute Seebeck values for junctions based on conjugated molecules such as the ones used here. The negative sign of these Seebeck values also makes them unique and a good starting point to quantitatively analyze the transport properties of these junctions.

**Figure 3:**
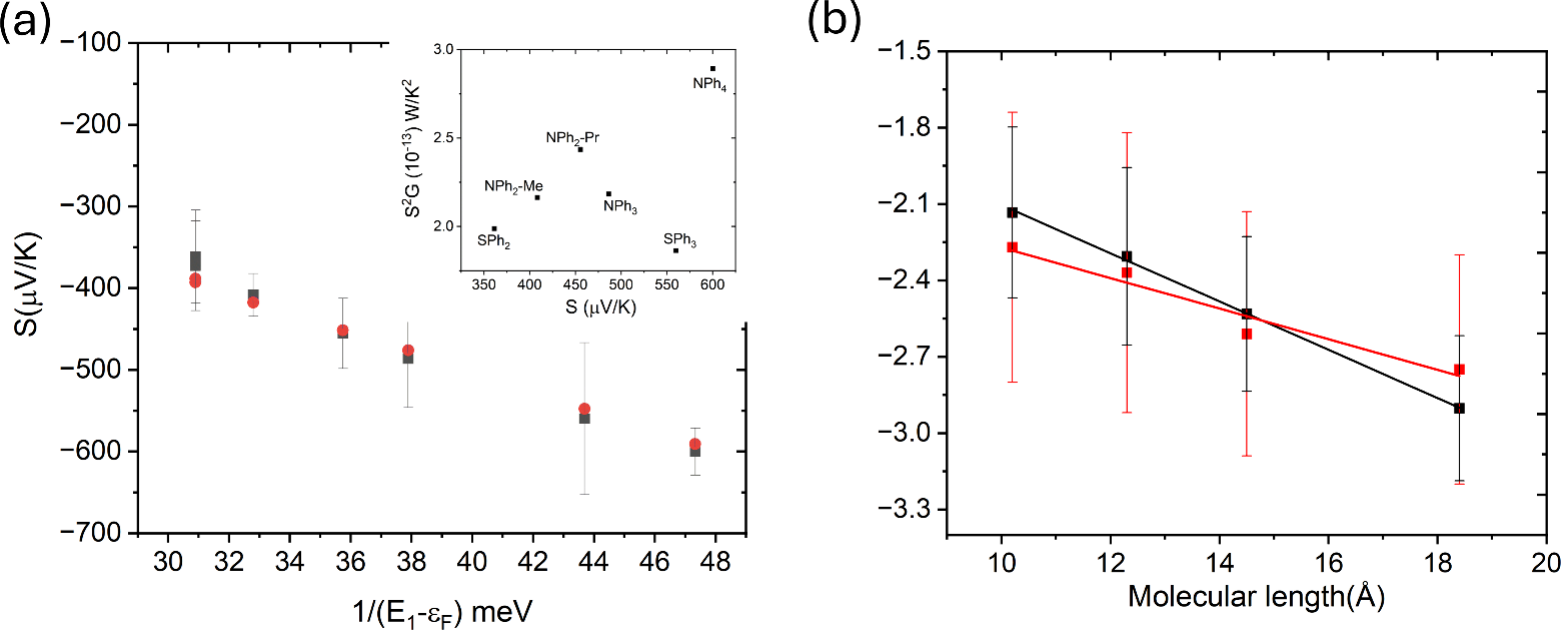
(a) Experimental (black) and theoretical (red) Seebeck values, plotted as a function of the energy position versus the Fermi energy of the quantized level, *E*_1_ (see text for details). Theoretical *S* values were calculated by eq 9 and *E*_1_ from eq 5. For each molecule, *S* values and their associated errors were calculated from the thermovoltage measurements in Figure 2c for each value of Δ*T.* The average of these *S* values, for each molecule, is reported in this figure along with the largest calculated error. Inset: The power factor plotted as a function of *S*. (b) The experimental conductance values of junctions based on the amino-terminated molecules (gray rectangles) and after correcting for the change in the interfacial DOS due to the quantized level (blue rectangles).

The negative sign of the resulting Seebeck values, see Figure 3a, indicates transport that is dominated by higher transmission of carriers above *ε*_*F*_ than below this level. This finding contrasts our previous thermovolatge measurements of junctions based on alkane chains on Bi^66–69^ and of junctions with conjugated molecules on other metals,^75–77^ in which a positive sign of *S* has been determined and interpreted as transport dominated by the HOMO levels. Naturally, this change of sign raises the question whether transport is governed here by their LUMOs.

We rule out this explanation by the following reasoning. Based on the onset of the UPS signals of the involved molecules adsorbed on Bi, prior to the formation of junctions (see Figure S3 in the SI), we determine the energy position of their HOMO levels versus *ε*_*F*_ to be Δ*E*_*HOMO*_^*N*^ ≅ 1.3eV and Δ*E*_*HOMO*_^*S*^ ≅ 1.0eV for the amine- and thiol-terminated monolayers, respectively. Since the HOMO–LUMO gap of these molecules (in the gas phase) is ∼4 eV, and since renormalization of this gap, due to interaction with the leads, is at the most on the order of ∼1 eV,^76,77^ the LUMO levels are expected to be ∼2.0 eV above *ε*_*F*_. Under such conditions, transport should still be HOMO-dominated and the sign of *S* should be positive and not negative, as measured.

This estimated value for the LUMO of the junctions contrasts with conductance peaks that appear in their d*I*/d*V* measurements and within the energy window defined by *∂f*/*∂E* at room temperature (Figure 2d). Such peaks, within a bias range of ±0.1 V, have never been observed in previous measurements of junctions with similar conjugated molecules. As their width is ∼20 meV, they cannot be associated with inelastic tunneling processes due to vibrational modes, as then their width at room temperature should have been ∼5.4*k_B_T* > 100 meV.^78^

These conductance features together with the above rationalization that rules out their molecular (LUMO) nature suggest that the TE properties of these junctions are governed by quantized levels within their Bi leads in a very similar way to the one discussed in one of our previous papers.^69^ However, there is an important difference: here the Seebeck value depends on only one free parameter, which is the length of the molecules. By varying this parameter, the potential drop across each junction is divided differently between the drop across the molecular layer and the built-in potential within the Bi. This, in turn, enables tuning of the energy position of the quantized (2D) interfacial states that, being sufficiently close to the Fermi level, substantially affect the TE properties (see Figure 1).

To quantitatively account for their effect (see eq 3), we start by estimating the overall potential drop in all junctions. The work function of all Bi films covered with the adsorbed monolayers studied here was determined by UPS to be 4.0 ± 0.1 eV. With an effective work function for (oxide covered) EGaIn of 4.5 eV, the potential drop in all junctions is therefore estimated to be *V* = 0.50 V. Details describing the calculation of the spatial distribution of this potential across the interface using a self-consistent Poisson equation can be found in the SI. Resulting curves from these calculations appear in Figure 4a. From the built-in potential curves in this figure we approximate, for simplicity, the length of the depletion layers within the Bi in all junctions to be *W* ≈ 40 nm. In the discussion below this value is justified. We further simplify the problem and treat the built-in potential as a triangular potential well (see inset in Figure 4b). All these simplifications make it possible to treat the transport properties analytically and, as is shown below, with excellent agreement with the experimental results.

**Figure 4:**
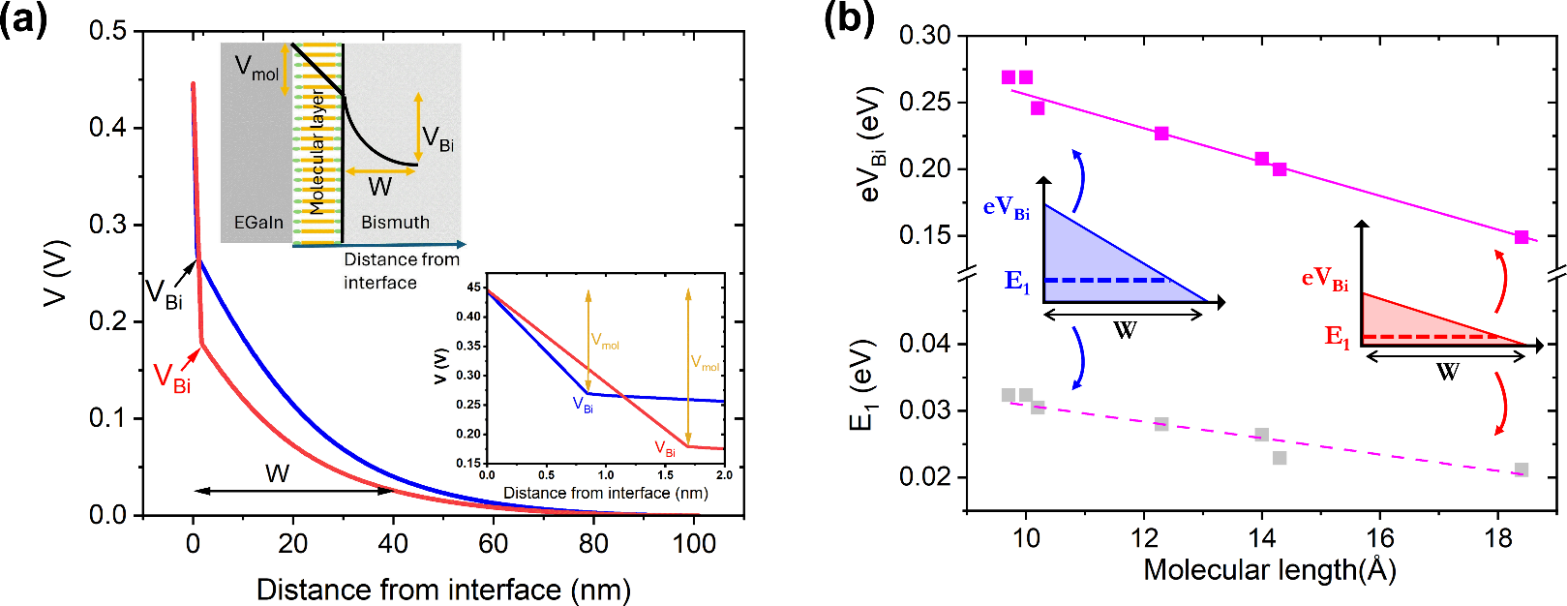
(a) Calculated potential distribution across junctions based on NPh_2_ (blue) and NPh_4_ (red) junctions. These two curves bracket the calculated curves for the rest of the molecules, which were omitted here for clarity. Zero is defined as the EGaIn surface. Top inset: a scheme of a junction depicting the various parameters used in the model. Lower inset: A zoom-in close to zero plotting the potential drop on the molecules and the edge of the built-in potential within the Bi. As the molecular length increases, *V*_*mol*_ increases and as a result *V*_*Bi*_ decreases. (b) The magnitude (in magenta points) of the potential wells formed by the depletion layers, *V*_*Bi*_, and the energy (in gray points) of the quantized level *E*_1_ (calculated by eq 5), plotted as a function of molecular length. The inset shows the triangular potential wells corresponding (coded with the same color) to the depletion layers in (a). They schematically show that as *L* becomes longer, *V*_*Bi*_ is smaller and consequently *E*_1_ is shifted closer to *ε*_*F*_.

Under the assumption of a triangular potential well, the Schrödinger equation gives Airy function solutions for the self-energies of the quantized states:

4 $$E_{n}={\left(\frac{\hbar^{2}}{2m^{*}}\right)}^{1/3}{\left(eF\right)}^{2/3}a_{n}$$

where m* is the effective mass (taken here for holes in Bi^79^ to be 0.65*m*_0_), *F* is the field within the well, and *a*_*n*_ are the zeros of the Airy function.

To calculate the depletion field, *F*, the metal–molecular layer–semimetal interface is treated as a series of capacitors with an (area-normalized) molecular capacitance *C*_*mol*_ = *ε*_*mol*_/*L* and a semimetal depletion capacitance *C*_*dep*_ = *ε*_*Bi*_/*W*, where *ε*_mol_ = 5 is the dielectric constant of the molecular layers.^79–81^

Since the displacement field is continuous *D* = *ε*_*mol*_ *E*_*mol*_ = *ε*_*sm*_ *F* (with *E*_*mol*_ as the field on the molecular layer) and the voltage across the interface is *V* = *V*_*mol*_ + *V*_*Bi*_, using *V*_*mol*_ = *DL*/*ε*_*mol*_ gives, for a given potential drop *V*,

5 $$E_{n}=a_{n}{\left(\frac{\hbar^{2}}{2m^{*}}\right)}^{1/3}{\left[e\frac{V}{L+\lambda }\frac{\varepsilon_{\mathrm{mol}}}{\varepsilon_{\mathrm{Bi}}}\right]}^{2/3}$$

where $\lambda =\frac{\varepsilon_{\mathrm{mol}}}{\varepsilon_{\mathrm{Bi}}}W$ . Equation 5 explicitly defines the electric field, *F*, in eq 4.

Note that since for all junctions *V*, which depends on the work function difference between the EGaIn and the monolayer-covered Bi, is identical (0.5 V) and *W* is also to a good approximation identical (40 nm), the self-energies within the potential wells depend on a single free parameter, the molecular length, *L*.

Plugging in numbers into eq 5, it turns out that the formed potential wells (see Figure 1 and also Figure 4a) accommodate in all junctions only one quantized level (see inset in Figure 4b), with energies *E*_1_ (relative to *ε*_*F*_ = 0) that depend solely on the molecular length *L*, as shown in Figure 4b. This can be understood in the following way: since *V* = *V*_*mol*_ + *V*_*Bi*_ is constant, with increasing *L* the potential drop on the molecules, *V*_*mol*_, increases and consequently the built-in potential within the Bi, *V*_*Bi*_, decreases. As this value is the depth of the confining potential well, with its decrease *E*_*1*_ is pushed toward the Fermi level, leading to higher transport asymmetry above and below this level, which results in higher value of *S*.

The values of *E*_1_ can be used to check the self-consistency of our simplifying assumption of using a constant depletion layer width of *W* ≈ 40 nm. For this purpose, we use the expected (based on the Poisson equation) 1D built-in potential within the Bi, which has the following form (see curves in Figure 4a):

6 $$V\left(x\right)=V_{Bi}\;\exp\left(-x/L_{SC}\right)$$

with *L*_*SC*_ as the space-charge length, defined by^67,68^

7 $$L_{SC}={\left[\frac{6\pi n_{0}e^{2}}{\varepsilon_{Bi}\varepsilon_{0}}\left(\frac{1}{\zeta_{h}}+\frac{1}{\zeta_{e}}\right)\right]}^{-1/2}$$

where the Fermi levels of the valence and conduction bands *ξ*_*h*_ and *ξ*_*e*_, are defined in Figure 1.

In response to a built-in potential, the edge of the conduction band is shifted above the Fermi level (see Figure 1) and becomes irrelevant for the calculation of *L*_*SC*_, while *ξ*_*h*_ progressively increases, being the energy difference between the (bulk) Fermi level and the shifted (up) edge of the valence band. Using *n*_0_ = 3.5 × 10^17^ cm^–3^ as the density of charge carriers in Bi^67,68^ and *ξ*_*h*_ = 0.25 eV (see Figures 1 and 4b) results in *L*_*SC*_ = 15 nm. With *eV*_*Bi*_ = *ξ*_*h*_, plugging in *E*_1_ = 0.02 eV as a typical value (see Figure 4b) for *eV*(*x*) in eq 6 gives *x* (that is an approximate value for *W*) on the order of 40 nm.

The analyze the effect of the quantized level in each junction on transport and Seebeck value, we invoke a Breit–Wigner expression to analyze the transmission (see Figure 1c):

8 $$\tau \left(E\right)\approx \frac{\Gamma_{M}\left(E\right)\Gamma_{Bi}}{{\left(E-E_{1}\right)}^{2}+{\left(\frac{\Gamma_{M}\left(E\right)+\Gamma_{Bi}}{2}\right)}^{2}}$$

where Γ_*Bi*_ is the coupling of the level to the bulk of Bi (assumed, for simplicity, to be energy independent), while the coupling to the metal lead (the EGaIn) depends on the potential barrier defined by the HOMO of each molecule:

9 $$\Gamma_{M}\left(E\right)=\Gamma_{0}\;e^{-\kappa \left(E\right)L}$$

where specifically $\kappa \left(E_{1}\right)=\sqrt{\frac{2m}{\hbar^{2}}\left|E_{1}-E_{HOMO}\right|}$ . We assume that the values for *E*_*HOMO*_ are the ones extracted from the UPS measurements and, as discussed above, the HOMO is closer than the LUMO to the Fermi energy.

Using the Mott formula (eq 1) and ignoring any energy dependency of Γ gives

10 $$S=-\frac{\pi^{2}k_{B}^{2}T}{3e}\left[\sqrt{\frac{m}{2\hbar^{2}}}\frac{L}{\sqrt{\varepsilon_{F}-E_{HOMO}}}-\frac{2\left(\varepsilon_{F}-E_{1}\right)}{{\left(\varepsilon_{F}-E_{1}\right)}^{2}+{\left(\Gamma /2\right)}^{2}}\right]$$

with Γ = Γ_*Bi*_ + Γ_*M*_.

Equation 10 gives a specific interpretation to the derivative expression of *A*(*E*) in eq 3. Values of *S* were calculated by this equation after plugging in values for *E*_*1*_ calculated by eq 5 for each molecular length. The very good agreement between these theoretical values and the experimental results shown in Figure 3b supports the suggested model of transport. It also supports both the self-consistency of the analytical analysis and the experimental concept presented here to tune the TE properties of molecular junctions.

Additional support for the suggested model can be found by examining the conductance behavior as a function of molecular length (Figure 3b). For this analysis only amino-terminated molecules are used with their identical Δ*E*_*HOMO*_^*N*^ value. The gray points in Figure 3b show the experimental results, which on a log scale appear to behave linearly with *L* with a slope value of −0.06 ± 0.01 Å^–1^. Judging by previous measurements of similar molecules that report a slope of 0.22 ± 0.01 Å^–1^ for a HOMO level positioned similarly to our junctions (∼1.0 eV below *ε*_*F*_ ^77^), the slope found here is small. The conductance hardly changes with *L*. This difference in slope values can be explained if the change in the DOS at the Bi lead as a function of molecular length is considered. With increasing molecular length, the quantized level is shifted closer to *ε*_*F*_ (Figure 4b) and as a result the conductance increases. Once this shift of *E*_1_, which depends on *L*, is accounted for by subtracting from the experimental results log­[(*ε_F_* – *E*_1_)^−2^] (see SI for more details), the fit to linear behavior with *L* becomes better (blue points in Figure 3b) and the slope becomes smaller, 0.15 ± 0.01 Å^–1^, in better agreement with previous results.

Note that both the evident agreement plotted in Figure 3a as a function of (*E*_1_ – *ε*_*F*_)^−1^ and the above correction to the conductance by log­[(*ε*_*F*_ – *E*_1_)^−2^ ] suggest that in eq 8, |*ε*_*F*_ – *E*_1_| ≫ Γ/2. Since Γ_*M*_ is expected to be small due to suppression by tunneling, the results essentially suggest that |*ε*_*F*_ – *E*_1_| ≫ Γ_*Bi*_. Since the quantized states are located within an order of ∼*k*_*B*_ *T* above *ε*_*F*_, this means that the coupling of the quantized levels formed within the potential wells to the bulk of Bi is small. Importantly, this implied small value does not contradict the apparent width of the quantized states as they appear in the d*I*/d*V* plots in Figure 1d. The width in these measurements is a result of the modulated bias voltage *V*_*ac*_ (with an amplitude of 20 mV) that has been applied in these measurements as part of the lock-in detection scheme.

The possible small value of Γ_*Bi*_ requires further attention that is beyond the scope of this paper. It is important, however, to comment that it appears to agree with conclusions drawn from a large experimental effort that has been made to study the topological properties of Bi films, in which metallic surface states have been identified with small coupling to the bulk of films.^82–91^ While in these previous studies small coupling was associated with an energy gap formed in the bulk of thin Bi films due to quantum confinement, here it most probably associated with the fact that a built-in potential exists at the interface. The value of Γ_*Bi*_ also determines the extent by which *E*_*1*_ can be pushed toward the Fermi level in a way that affects *S*. Once this resonant level resides within Γ_*Bi*_ from the Fermi level, its effect on *S* becomes negligible.

The inset in Figure 3a plots the power factor of the junctions as a function of *S*, which is another way to appreciate the dual role the interfacial resonant level in each junction has affecting both *S* and *G*.

Before concluding we note that this work essentially offers a glimpse into the potentially rich playground of various effects that molecular layers ensembled on semimetals can induce. Future work, for example, needs to explore the combination of the effects described here using conjugated molecules with smaller HOMO–LUMO gaps in order to further improve the power factor, *S* ^2^ *G*. The implications of this method as a tool to form 2D-confined carriers subjected to a Rashba effect are also noted and deserve further attention.

## Supplementary Material



## Notes

Additional experimental and characterization details and calculation method for the built-in potentials (PDF)

## References

[1] Claughton N. R., Lambert C. J. (1996). Thermoelectric Properties of Mesoscopic Superconductors. Phys. Rev. B.

[2] Brandner K., Saito K., Seifert U. (2013). Strong Bounds on Onsager
Coefficients
and Efficiency for Three-Terminal Thermoelectric Transport in a Magnetic
Field. Phys. Rev. Lett..

[3] Marchegiani G., Virtanen P., Giazotto F., Campisi M. (2016). Self-Oscillating Josephson
Quantum Heat Engine. Phys. Rev. Appl..

[4] Kamp M., Sothmann B. (2019). Phase-Dependent Heat
and Charge Transport through Superconductor-Quantum
Dot Hybrids. Phys. Rev. B.

[5] Sothmann B., Sánchez R., Jordan A. N. (2015). Thermoelectric Energy
Harvesting
with Quantum Dots. Nanotechnology.

[6] Ozaeta A., Virtanen P., Bergeret F. S., Heikkilä T. T. (2014). Predicted
Very Large Thermoelectric Effect in Ferromagnet-Superconductor Junctions
in the Presence of a Spin-Splitting Magnetic Field. Phys. Rev. Lett..

[7] Esposito M., Lindenberg K., Van den Broeck C. (2009). Thermoelectric Efficiency at Maximum
Power in a Quantum Dot. Europhys. Lett..

[8] Cui L., Miao R., Wang K., Thompson D., Zotti L. A., Cuevas J. C., Meyhofer E., Reddy P. (2018). Peltier Cooling in
Molecular Junctions. Nat. Nanotechnol..

[9] Whitney R. S. (2014). Most Efficient
Quantum Thermoelectric at Finite Power Output. Phys. Rev. Lett..

[10] Ofarim A., Kopp B., Möller T., Martin L., Boneberg J., Leiderer P., Scheer E. (2016). Thermo-Voltage Measurements of Atomic
Contacts at Low Temperature. Beilstein J. Nanotechnol..

[11] Ronetti F., Vannucci L., Dolcetto G., Carrega M., Sassetti M. (2016). Spin-Thermoelectric
Transport Induced by Interactions and Spin-Flip Processes in Two-Dimensional
Topological Insulators. Phys. Rev. B.

[12] Giazotto F., Robinson J. W. A., Moodera J. S., Bergeret F. S. (2014). Proposal for a Phase-Coherent
Thermoelectric Transistor. Appl. Phys. Lett..

[13] Azema J., Lombardo P., Daré A.-M. (2014). Conditions
for Requiring Nonlinear
Thermoelectric Transport Theory in Nanodevices. Phys. Rev. B.

[14] Kim Y., Jeong W., Kim K., Lee W., Reddy P. (2014). Electrostatic
Control of Thermoelectricity in Molecular Junctions. Nat. Nanotechnol..

[15] Zimbovskaya N. A. (2015). The Effect
of Coulomb Interactions on Nonlinear Thermovoltage and Thermocurrent
in Quantum Dots. J. Chem. Phys..

[16] Svilans A., Burke A. M., Svensson S. F., Leijnse M., Linke H. (2016). Nonlinear
Thermoelectric Response Due to Energy-Dependent Transport Properties
of a Quantum Dot. Phys. Low-dimensional Syst.
Nanostructures.

[17] Sánchez D., Serra L. (2011). Thermoelectric Transport of Mesoscopic
Conductors Coupled to Voltage
and Thermal Probes. Phys. Rev. B.

[18] Boese D., Fazio R. (2001). Thermoelectric Effects
in Kondo-Correlated Quantum Dots. Europhys.
Lett..

[19] Sánchez D., López R. (2016). Nonlinear Phenomena in Quantum Thermoelectrics and
Heat. Comptes Rendus. Phys..

[20] Whitney R. S. (2013). Thermodynamic
and Quantum Bounds on Nonlinear Dc Thermoelectric Transport. Phys. Rev. B.

[21] Erdman P. A., Peltonen J. T., Bhandari B., Dutta B., Courtois H., Fazio R., Taddei F., Pekola J. P. (2019). Nonlinear Thermovoltage
in a Single-Electron Transistor. Phys. Rev.
B.

[22] Hwang S.-Y., López R., Sánchez D. (2015). Cross Thermoelectric Coupling in
Normal-Superconductor Quantum Dots. Phys. Rev.
B.

[23] Shi X.-L., Li N.-H., Li M., Chen Z.-G. (2025). Toward Efficient
Thermoelectric Materials and Devices: Advances, Challenges, and Opportunities. Chem. Rev..

[24] Mazzacua A., Giulio F., Narducci D. (2026). Charge Carrier Extrinsic
Energy Filtering:
Theoretical Advances, Applications, and Perspectives in Thermoelectricity. Chem. Phys. Rev..

[25] Narducci D. (2019). Thermoelectric
Harvesters and the Internet of Things: Technological and Economic
Drivers. J. Phys. Energy.

[26] Gemma A., Gotsmann B. (2021). A Roadmap for Molecular Thermoelectricity. Nat. Nanotechnol..

[27] Korol R., Kilgour M., Segal D. (2016). Thermopower of Molecular
Junctions:
Tunneling to Hopping Crossover in DNA. J. Chem.
Phys..

[28] Hicks L. D., Dresselhaus M. S. (1993). Effect
of Quantum-Well Structures on the Thermoelectric
Figure of Merit. Phys. Rev. B.

[29] Hicks L. D., Dresselhaus M. S. (1993). Thermoelectric
Figure of Merit of a One-Dimensional
Conductor. Phys. Rev. B.

[30] Lan Y., Minnich A. J., Chen G., Ren Z. (2010). Enhancement of Thermoelectric
Figure-of-Merit by a Bulk Nanostructuring Approach. Adv. Funct. Mater..

[31] Zebarjadi M., Joshi G., Zhu G., Yu B., Minnich A., Lan Y., Wang X., Dresselhaus M., Ren Z., Chen G. (2011). Power Factor
Enhancement by Modulation Doping in Bulk Nanocomposites. Nano Lett..

[32] Narducci D., Zulian L., Lorenzi B., Giulio F., Villa E. (2021). Exceptional
Thermoelectric Power Factors in Hyperdoped, Fully Dehydrogenated Nanocrystalline
Silicon Thin Films. Appl. Phys. Lett..

[33] Mao J., Liu Z., Ren Z. (2016). Size Effect
in Thermoelectric Materials. npj Quantum Mater..

[34] Heremans J. P., Wiendlocha B., Chamoire A. M. (2012). Resonant Levels in Bulk Thermoelectric
Semiconductors. Energy Environ. Sci..

[35] Heremans J. P., Jovovic V., Toberer E. S., Saramat A., Kurosaki K., Charoenphakdee A., Yamanaka S., Snyder G. J. (2008). Enhancement of Thermoelectric
Efficiency in PbTe by Distortion of the Electronic Density of States. Science.

[36] Sakane S., Ishibe T., Mizuta K., Fujita T., Kiyofuji Y., Ohe J. I., Kobayashi E., Nakamura Y. (2021). Anomalous Enhancement
of Thermoelectric Power Factor by Thermal Management with Resonant
Level Effect. J. Mater. Chem. A.

[37] Hung N. T., Hasdeo E. H., Nugraha A. R. T., Dresselhaus M. S., Saito R. (2016). Quantum Effects in
the Thermoelectric Power Factor of Low-Dimensional
Semiconductors. Phys. Rev. Lett..

[38] Ohta H., Kim S. W., Kaneki S., Yamamoto A., Hashizume T. (2018). High Thermoelectric
Power Factor of High-Mobility 2D Electron Gas. Adv. Sci..

[39] Uematsu Y., Ishibe T., Mano T., Ohtake A., Miyazaki H. T., Kasaya T., Nakamura Y. (2024). Anomalous
Enhancement of Thermoelectric
Power Factor in Multiple Two-Dimensional Electron Gas System. Nat. Commun..

[40] Uematsu Y., Ishibe T., Kozuki S., Mano T., Ohtake A., Miyazaki H. T., Kasaya T., Yamashita Y., Uenuma M., Nakamura Y. (2024). Film Thermoelectric Generator of
Multiple 2D Electron Gas. IEEE Trans. Electron
Devices.

[41] Heremans J. P., Thrush C. M., Morelli D. T., Wu M.-C. (2002). Thermoelectric Power
of Bismuth Nanocomposites. Phys. Rev. Lett..

[42] Li P., Selzer Y. (2024). Disordered Ballistic
Bismuth Nano-waveguides for Highly
Efficient Thermoelectric Energy Conversion. Small.

[43] Shapira E., Holtzman A., Marchak D., Selzer Y. (2012). Very High Thermopower
of Bi Nanowires with Embedded Quantum Point Contacts. Nano Lett..

[44] Han F., Andrejevic N., Nguyen T., Kozii V., Nguyen Q. T., Hogan T., Ding Z., Pablo-Pedro R., Parjan S., Skinner B., Alatas A., Alp E., Chi S., Fernandez-Baca J., Huang S., Fu L., Li M. (2020). Quantized Thermoelectric Hall Effect Induces Giant Power Factor in
a Topological Semimetal. Nat. Commun..

[45] Michibata A., Terada T., Matsuzono K., Ishibe T., Yamashita Y., Naruse N., Suzuki K., Nakamura Y. (2026). Boosting Seebeck Coefficient
through Electron-Phonon Interaction by Phonon Frequency Control. Phys. Rev. Mater..

[46] Miao R., Xu H., Skripnik M., Cui L., Wang K., Pedersen K. G. L., Leijnse M., Pauly F., Wärnmark K., Meyhofer E., Reddy P., Linke H. (2018). Influence
of Quantum
Interference on the Thermoelectric Properties of Molecular Junctions. Nano Lett..

[47] Arroyo C. R., Tarkuc S., Frisenda R., Seldenthuis J. S., Woerde C. H. M., Eelkema R., Grozema F. C., van der
Zant H. S. J. (2013). Signatures of Quantum Interference Effects on Charge
Transport Through a Single Benzene Ring. Angew.
Chem..

[48] Nozaki D., Avdoshenko S. M., Sevinçli H., Cuniberti G. (2014). Quantum Interference
in Thermoelectric Molecular Junctions: A Toy Model Perspective. J. Appl. Phys..

[49] Garner M.
H., Li H., Chen Y., Su T. A., Shangguan Z., Paley D. W., Liu T., Ng F., Li H., Xiao S., Nuckolls C., Venkataraman L., Solomon G. C. (2018). Comprehensive Suppression of Single-Molecule Conductance
Using Destructive σ-Interference. Nature.

[50] Kaliginedi V., Moreno-García P., Valkenier H., Hong W., García-Suárez V. M., Buiter P., Otten J. L. H., Hummelen J. C., Lambert C. J., Wandlowski T. (2012). Correlations between Molecular Structure and Single-Junction
Conductance: A Case Study with Oligo­(Phenylene-Ethynylene)-Type Wires. J. Am. Chem. Soc..

[51] Lambert C. J. (2015). Basic Concepts
of Quantum Interference and Electron Transport in Single-Molecule
Electronics. Chem. Soc. Rev..

[52] Finch C. M., García-Suárez V. M., Lambert C. J. (2009). Giant Thermopower
and Figure of Merit in Single-Molecule Devices. Phys. Rev. B.

[53] Karlström O., Linke H., Karlström G., Wacker A. (2011). Increasing Thermoelectric
Performance Using Coherent Transport. Phys.
Rev. B.

[54] Bergfield J. P., Solis M. A., Stafford C. A. (2010). Giant Thermoelectric
Effect from
Transmission Supernodes. ACS Nano.

[55] Keane Z. K., Ciszek J. W., Tour J. M., Natelson D. (2006). Three-Terminal Devices
to Examine Single-Molecule Conductance Switching. Nano Lett..

[56] Sánchez D., López R. (2013). Scattering
Theory of Nonlinear Thermoelectric Transport. Phys. Rev. Lett..

[57] Nakpathomkun N., Xu H. Q., Linke H. (2010). Thermoelectric Efficiency at Maximum
Power in Low-Dimensional Systems. Phys. Rev.
B.

[58] Gehring P., Sowa J. K., Hsu C., de Bruijckere J., van der Star M., Le Roy J. J., Bogani L., Gauger E. M., van der Zant H. S. J. (2021). Complete Mapping of the Thermoelectric
Properties of
a Single Molecule. Nat. Nanotechnol..

[59] Ballmann S., Härtle R., Coto P. B., Elbing M., Mayor M., Bryce M. R., Thoss M., Weber H. B. (2012). Experimental Evidence
for Quantum Interference and Vibrationally Induced Decoherence in
Single-Molecule Junctions. Phys. Rev. Lett..

[60] He P., Daaoub A. H. S., Sangtarash S., Sadeghi H., Yoon H. J. (2024). Thermopower
in Underpotential Deposition-Based Molecular Junctions. Nano Lett..

[61] Gu M.-W., Peng H. H., Chen I.-W. P., Chen C. (2021). Tuning Surface
d Bands
with Bimetallic Electrodes to Facilitate Electron Transport across
Molecular Junctions. Nat. Mater..

[62] Jang J., Tanaka Y., Lee D., He P., Ohto T., Kim Z. H., Yoon H. J. (2026). Concurrent Enhancement
of Thermopower
and Conductivity via Modulation of Diacetylide-Electrode Coupling
in Molecular Junctions. Nano Lett..

[63] Newns D. M. (1969). Self-Consistent
Model of Hydrogen Chemisorption. Phys. Rev..

[64] Hammer B., Norskov J. K. (1995). Why Gold Is the
Noblest of All the Metals. Nature.

[65] Gu M., Lai C., Ni I., Wu C., Chen C. (2023). Increased Surface Density
of States at the Fermi Level for Electron Transport Across Single-Molecule
Junctions. Angew. Chemie Int. Ed..

[66] Li P., Selzer Y. (2022). Molecular Ensemble
Junctions with Inter-Molecular Quantum
Interference. Nat. Commun..

[67] Shmueli S., Cohen Jungerman M., Shekhter P., Selzer Y. (2024). Efficient
Molecular
Rectification in Metal-Molecules-Semimetal Junctions. J. Phys. Chem. Lett..

[68] Frank T., Shmueli S., Cohen Jungerman M., Shekhter P., Selzer Y. (2023). Large Seebeck
Values in Metal-Molecule-Semimetal Junctions Attained by a Gateless
Level-Alignment Method. Nano Lett..

[69] Cohen
Jungerman M., Shmueli S., Shekhter P., Selzer Y. (2025). Unusually
High Thermopower in Molecular Junctions from Molecularly Induced Quantized
States in Their Semimetal Leads. Nano Lett..

[70] Toudert J., Serna R., Deeb C., Rebollar E. (2019). Optical Properties
of Bismuth Nanostructures towards the Ultrathin Film Regime. Opt. Mater. Express.

[71] Li X., Wei Y., Lu G., Mei Z., Zhang G., Liang L., Li Q., Fan S., Zhang Y. (2022). Gate-Tunable Contact-Induced Fermi-Level
Shift in Semimetal. Proc. Natl. Acad. Sci. U.
S. A..

[72] Hofmann P. (2006). The Surfaces
of Bismuth: Structural and Electronic Properties. Prog. Surf. Sci..

[73] Verdun H. R. (1974). Inter-Magnetic-Subband
Excitons in Bismuth. Phys. Rev. Lett..

[74] Isaacson R. T., Williams G. A. (1969). Alfvén-Wave
Propagation in Solid-Stae Plasmas.
III. Quantum Oscillations of the Fermi Surface of Bismuth. Phys. Rev..

[75] Jang J., He P., Yoon H. J. (2023). Molecular Thermoelectricity in EGaIn-Based Molecular
Junctions. Acc. Chem. Res..

[76] Tan A., Sadat S., Reddy P. (2010). Measurement
of Thermopower and Current-Voltage
Characteristics of Molecular Junctions to Identify Orbital Alignment. Appl. Phys. Lett..

[77] Tan A., Balachandran J., Dunietz B. D., Jang S.-Y., Gavini V., Reddy P. (2012). Length Dependence
of Frontier Orbital Alignment in Aromatic Molecular
Junctions. Appl. Phys. Lett..

[78] Galperin M., Ratner M. A., Nitzan A. (2004). Inelastic Electron Tunneling Spectroscopy
in Molecular Junctions: Peaks and Dips. J. Chem.
Phys..

[79] Sun X., Zhang Z., Dresselhaus M. S. (1999). Theoretical
Modeling of Thermoelectricity
in Bi Nanowires. Appl. Phys. Lett..

[80] Van
Dyck C., Marks T. J., Ratner M. A. (2017). Chain Length Dependence of the Dielectric
Constant and Polarizability in Conjugated Organic Thin Films. ACS Nano.

[81] Heitzer H. M., Marks T. J., Ratner M. A. (2014). Maximizing the Dielectric Response
of Molecular Thin Films via Quantum Chemical Design. ACS Nano.

[82] Hirahara T., Nagao T., Matsuda I., Bihlmayer G., Chulkov E. V., Koroteev Y. M., Echenique P. M., Saito M., Hasegawa S. (2006). Role of Spin-Orbit Coupling and Hybridization
Effects in the Electronic Structure of Ultrathin Bi Films. Phys. Rev. Lett..

[83] Jnawali G., Klein C., Wagner T., Hattab H., Zahl P., Acharya D. P., Sutter P., Lorke A., Horn-von
Hoegen M. (2012). Manipulation of Electronic Transport in the Bi(111) Surface State. Phys. Rev. Lett..

[84] Xiao S., Wei D., Jin X. (2012). Bi­(111) Thin Film with Insulating Interior but Metallic
Surfaces. Phys. Rev. Lett..

[85] Kröger P., Abdelbarey D., Siemens M., Lükermann D., Sologub S., Pfnür H., Tegenkamp C. (2018). Controlling
Conductivity by Quantum Well States in Ultrathin Bi(111) Films. Phys. Rev. B.

[86] Aitani M., Hirahara T., Ichinokura S., Hanaduka M., Shin D., Hasegawa S. (2014). In Situ Magnetotransport Measurements in Ultrathin
Bi Films: Evidence for Surface-Bulk Coherent Transport. Phys. Rev. Lett..

[87] Zhu K., Wu L., Gong X., Xiao S., Jin X. (2016). Quantum Transport in
the Surface States of Epitaxial Bi(111) Thin Films. Phys. Rev. B.

[88] Hirahara T., Shirai T., Hajiri T., Matsunami M., Tanaka K., Kimura S., Hasegawa S., Kobayashi K. (2015). Role of Quantum
and Surface-State Effects in the Bulk Fermi-Level Position of Ultrathin
Bi Films. Phys. Rev. Lett..

[89] Hussain N., Liang T., Zhang Q., Anwar T., Huang Y., Lang J., Huang K., Wu H. (2017). Ultrathin Bi Nanosheets
with Superior Photoluminescence. Small.

[90] Yue D., Wang H., Huang G., Jiang Y., Huang Z., Zheng P., Song Y., Guo S., Tian N., Luo M., Guo Z., Luo H., Xi C., Kuang G., Watanabe K., Taniguchi T., Chen Z., Lin X., Wang J., Zheng C., Jin X., Ruan W., Zhang Y. (2025). Quantum Transport in Bismuth Two-Dimensional
Electron System. Phys. Rev. X.

[91] Bihlmayer G., Noël P., Vyalikh D. V., Chulkov E. V., Manchon A. (2022). Rashba-like
Physics in Condensed Matter. Nat. Rev. Phys..

