# Supplementary material for: Tuning of Thermopower in Molecular Junctions by Molecularly Controlled Sculpting of the Density of States in Their Leads
Source: Nano Lett. 2026 Jul 20;26(29):9589–97. doi: 10.1021/acs.nanolett.6c02296 (PMC13430674; doi:10.1021/acs.nanolett.6c02296)
Supplement: Supplementary file 1 [file nl6c02296_si_001.pdf]

Supporting Information

Tuning of thermopower in molecular junctions by molecularly controlled sculpting of the density of states in their leads

Mor Cohen Jungerman[1], Shachar Shmueli[1], Pini Shekhter[2], Yoram Selzer[1]*

[1]Department of Chemical Physics, School of Chemistry, Tel Aviv University, Tel Aviv 69978, Israel.

[2]The Tel Aviv Center for Nanoscience and Nanotechnology, Tel Aviv 69978, Israel.

Formation and initial characterization of monolayers .

Layers of 300nm of Bi were thermally deposited onto of p-doped silicon (100) substrates with a silicon oxide (SiO_2) layer. Ultra-smooth Bi surfaces were subsequently prepared by a template-stripping process[1–3]. These template-stripped Bi films were used as the bottom lead in all junctions. Formation of monolayers, devoid of any remnants of native oxide of Bi, requires an incubation of 3 hours assembly in toluene solutions of the various conjugated molecules under dry conditions inside a glovebox. The necessary concentration of conjugated molecules to form high quality layers was found to be ~1mM. In the case for SPh_3, uniform layers are obtained already at concentration of 0.1mM, yielding water contact angles similar to previous results (Figure S1a)[4–8]. Contact angle measurements of conjugated molecules indicate that well-packed and ordered SAMs exhibit contact angles ≥85°[4–8]. For the other molecules in this study, 1mM assembly solutions were used to avoid low-solubility effects at higher concentrations for the longer chains. Indeed, as shown in Figure S1b, these assembly conditions yield ordered layers for all tested conjugated molecules, with contact angle ≥85°.

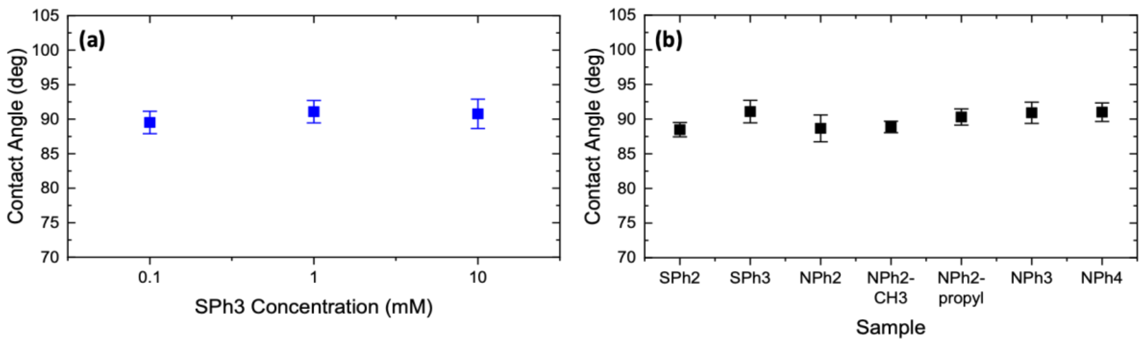

Figure S1. (a) Contact angle measurements of SPh_3 monolayers assembled from Toluene solutions with varying concentrations spanning two orders of magnitude. (b) The contact angle of all measured conjugated molecules assembled from 1mM solutions in Toluene.

The uniformity of the layers was also determined by XPS measurements to verify the removal of native oxide and the exchange of oxygen-bonded Bi by thiol/amine-bonded Bi on the surface. For this purpose, XPS measurements were collected in the regime of the S_{2s} orbital for thiol-based monolayers and the N_{1s} orbital for amine-based monolayers. Figure S2 shows that indeed with the concentration of ~1mM there are no Bi-O-S / Bi-O-N bonds on the surface and instead all surface Bi is connected to thiol/amine (Bi-S/Bi-N)[1–3].

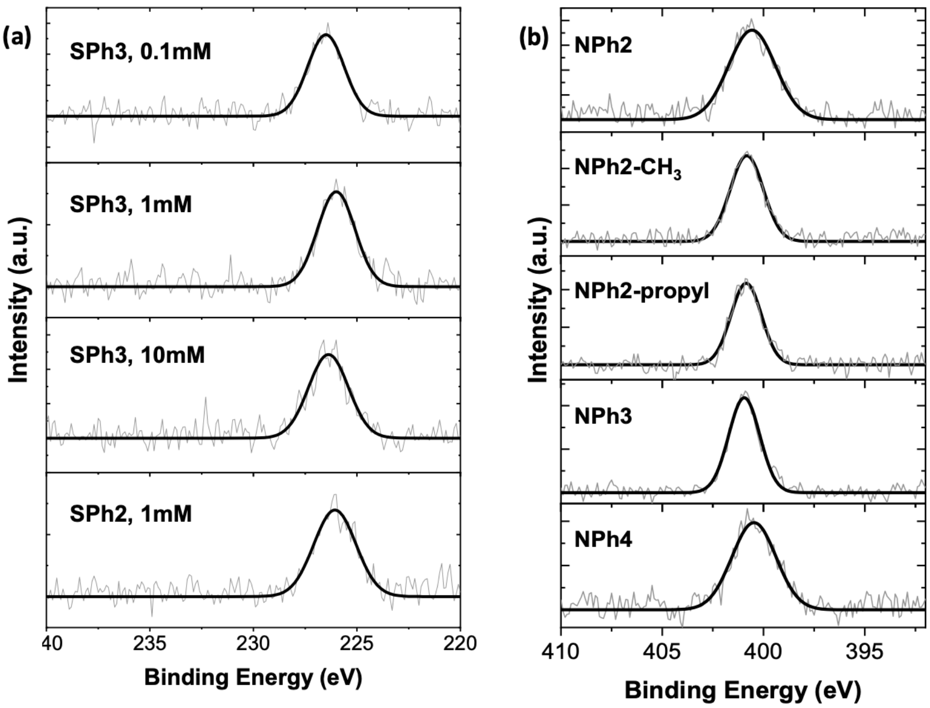

Figure S 2. XPS S_{2s} and N_{1s} spectra of the thiol and amine based molecules, respectively. (a) S_{2s} spectra for phenylthiol molecules incubated in toluene solutions of SPh_3 with concentrations of 0.1, 1, and 10 mM, and SPh_2 at 1 mM. (b) N_{1s} spectra for phenylamine molecules incubated in 1 mM toluene solutions.

Ultraviolet Photoelectron Spectroscopy (UPS) measurements .

UPS measurements were performed with He I (hν=21.22 eV) radiation and a 10V bias applied to the samples to improve the transmission of low kinetic energy (KE) electrons and to improve the determination of energy edges. Properties of bare Bi were also determined in some samples by Ar^+ ion sputtering at 1keV (with an estimated rate of 0.1 nm/sec) to clean them from organic contaminations and oxide. Figure S3 presents UPS results with a zoom-in on the photoemission onset, while Figure S4 shows a zoom-in on the photoemission cutoff. Together, these figures display, for all samples, both the Fermi level–HOMO gap, determined from the photoemission onset, and the work function, extracted from the photoemission cutoff.

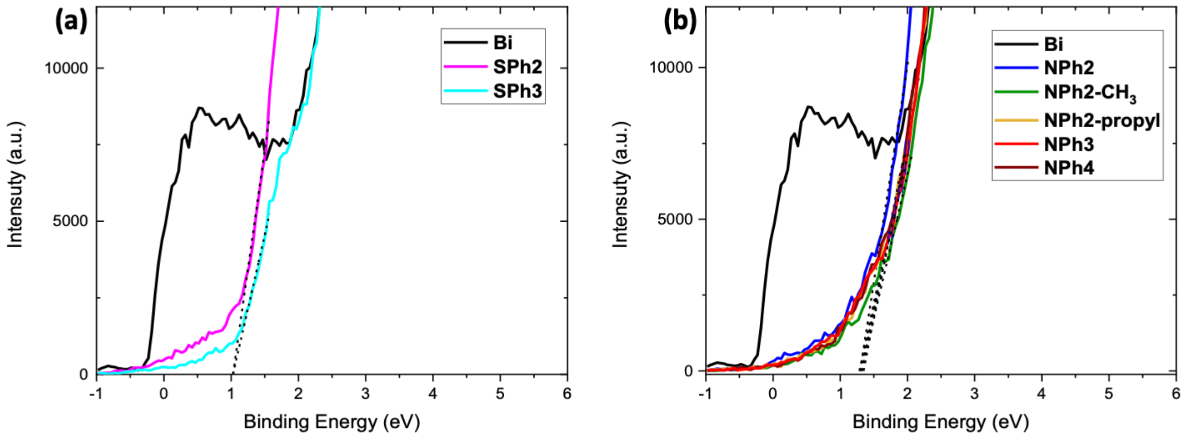

Figure S3. The photoemission onset of all monolayers investigated in this study compared to the onset of bare Bi (after stripping of the oxide layer via sputtering): (a) phenyl-thiols and (b) phenyl-amines.

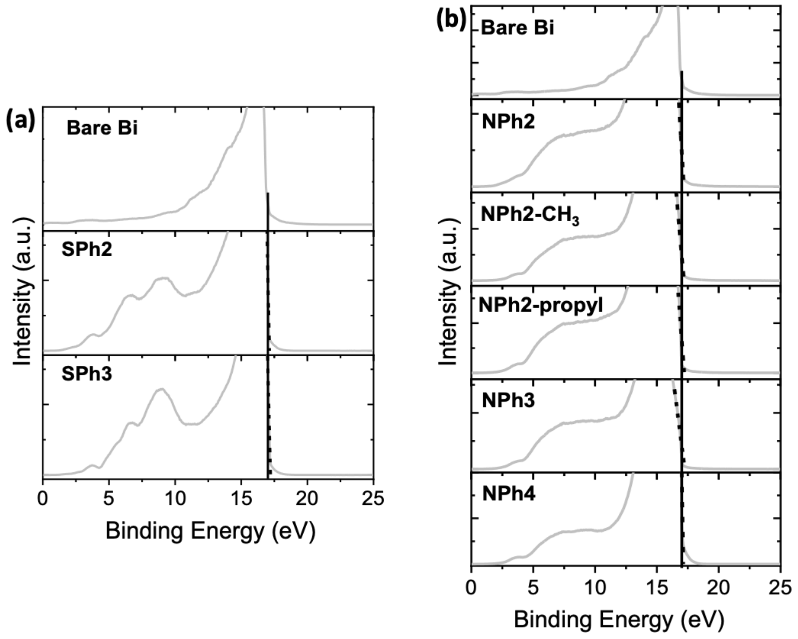

Figure S4. The photoemission cutoff for all monolayers investigated in this study compared to the onset of bare Bi (after stripping of the oxide layer via sputtering): (a) phenyl-thiols and (b) phenyl-amines.

Self-Consistent Electrostatic Model of a Metal–Insulator–Semimetal Junction

We consider a one-dimensional metal–insulator–semimetal (M–I–SM) junction. The electrostatic potential $\psi(x)$ is obtained by solving Poisson's equation:

$$\frac{d}{dx}\left[\varepsilon(x)\frac{d\psi(x)}{dx}\right] = \rho(x),$$

where $\varepsilon(x)$is the position-dependent dielectric permittivity and $\rho(x)$is the charge density. The dielectric profile is piecewise constant, corresponding to the insulating and semimetal regions.

The boundary condition at the metal interface is set by the work function difference:

$$\psi(0) = \Delta E = \phi_{\text{metal}} - \phi_{\text{semi}},$$

while at the far end of the semimetal a zero-field condition is imposed:

$$\frac{d\psi}{dx}|_{x=L} = 0.$$

The semimetal is modeled using overlapping conduction and valence bands. The band edges shift with the electrostatic potential:

$$E_c(x) = E_{c0} + \psi(x), E_v(x) = E_{v0} + \psi(x),$$

where $E_{c0}$and E_{v0}define the bulk band overlap.

Electron and hole densities are computed using a three-dimensional density-of-states model:

$$n(x) = N_c F_{1/2}(\eta_n), p(x) = N_v F_{1/2}(\eta_p),$$

with reduced energies

$$\eta_n = \frac{-E_c(x)}{k_B T}, \eta_p = \frac{E_v(x)}{k_B T}.$$

The effective density-of-states parameters are

$$N_c = g_e\, 2\left(\frac{2\pi m_e^* k_B T}{h^2}\right)^{3/2}, N_v = g_h\, 2\left(\frac{2\pi m_h^* k_B T}{h^2}\right)^{3/2},$$

where $m_e^*$and $m_h^*$are the electron and hole density-of-states effective masses, and g_e, $g_h$are degeneracy factors.

The Fermi–Dirac integral of order 1/2 is approximated as

$$F_{1/2}(\eta) \approx \frac{e^\eta}{1 + 0.27\, e^\eta},$$

which provides an accurate interpolation between non-degenerate and degenerate regimes.

The charge density is then given by

$$\rho(x) = q\,[p(x) - n(x)],$$

and vanishes in the insulating region.

Poisson's equation is discretized on a uniform grid using a finite-difference scheme that explicitly accounts for the spatial variation of the dielectric constant:

$$\frac{d}{dx}\left(\varepsilon\frac{d\psi}{dx}\right) \rightarrow \frac{\varepsilon_{i+1/2}(\psi_{i+1}-\psi_i)-\varepsilon_{i-1/2}(\psi_i-\psi_{i-1})}{\Delta x^2},$$

with $\varepsilon_{i\pm 1/2} = (\varepsilon_i + \varepsilon_{i\pm 1})/2$.

The resulting nonlinear system is solved self-consistently using a Newton–Raphson scheme:

$$\mathbf{J}\,\delta\psi = \mathbf{R},$$

where $\mathbf{R}$ is the discretized Poisson residual and $\mathbf{J}$ is the Jacobian matrix. The latter includes both electrostatic contributions and the derivative of the charge density:

$$\frac{d\rho}{d\psi} = q\left(\frac{dp}{d\psi} - \frac{dn}{d\psi}\right).$$

A damping factor is introduced to ensure numerical stability:

$$\psi^{(k+1)} = \psi^{(k)} - \alpha\,\delta\psi,$$

with $\alpha < 1$.

The initial potential profile is chosen to reflect the expected physical behavior: a linear drop across the insulator and an exponential decay into the semimetal, with a characteristic screening length

$$\lambda = \sqrt{\frac{\varepsilon_{\text{semi}} k_B T}{q^2(n_{\text{bulk}} + p_{\text{bulk}})}}.$$

Iterations are continued until the maximum residual falls below a prescribed tolerance.

Upon convergence, the conduction and valence band profiles are obtained from

$$E_c(x) = E_{c0} + \psi(x), E_v(x) = E_{v0} + \psi(x),$$

with the Fermi level taken as the reference energy. These profiles directly yield the band bending and depletion characteristics of the junction. Representative curves of $E_v(x)$ are presented in the main text.

The potential divider at the interface between the molecular layer and the built-in potential within the Bi critically depends on the (relative) dielectric constant of the molecules (see equation 4 in the main text). Figure S5 shows the effect of this dependency by presenting the potential calculations for two molecular layers with the same length (1.84nm, which corresponds to the length of NPh_4) but with two dielectric constants ε_{mol} =2 and ε_{mol} =5, that correspond to that of alkane and phenyl layers, respectively. While for $\varepsilon_{mol} = 5$, a quantized state can be formed within the confining potential (see Figure 4 in the main text), the potential well with $\varepsilon_{mol} = 2$, is too small for confinement.

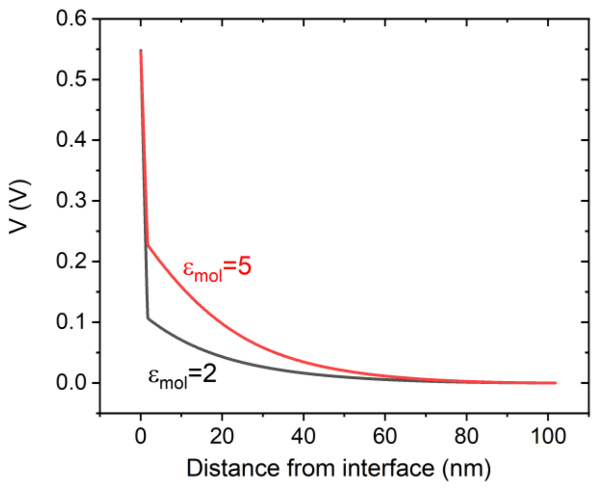

Figure S5. The effect of the molecular dielectric constant on the built-in potential within the Bi.

Effect of interfacial quantized state on conductance as a function of molecular length

The corrected behavior of conductance as a function of L in Figure 3, which accounts for the quantized interfacial state, uses the logarithm of the Breit-Wigner equation under the following conditions: $E_F - E_1 \gg \Gamma_M, \Gamma_{Bi}$. This leads from:

$$\tau(E) \approx \frac{\Gamma_{Bi}\Gamma_0 e^{-\kappa(E)L}}{(E_F - E_1)^2 + \left(\frac{\Gamma_M(E) + \Gamma_{Bi}}{2}\right)^2}$$

to:

$$\log \tau \approx -\kappa L + \log \Gamma_{Bi}\Gamma_0 + \log(E_F - E_1)^{-2}$$

With increasing L, E_1 is shifted closer to the Fermi level making the last log expression on the right larger. This increase compensates for the decrease in conductance due to the $-\kappa L$ contribution. In the main text,

to compare with previous reported values of slopes (κ), which were measured in systems without variation in the DOS of the leads but does exist in our junctions, the $\log(E_F - E_1)^{-2}$expression must be subtracted from the experimental results as it represents the changes in DOS. This subtraction results in the change of effective slope (κ) in Figure 3b.

The power factor of the junctions.

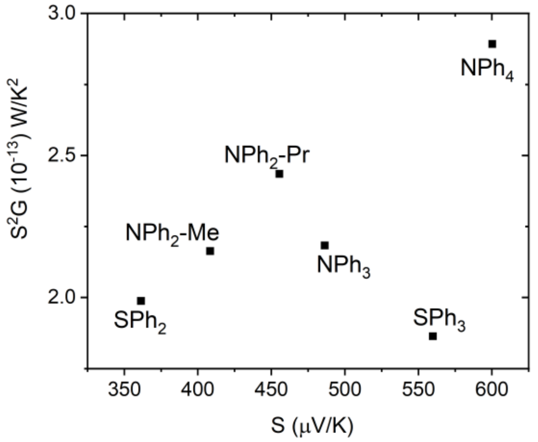

Figure S6. The power factor of the junctions as a function of their Seebeck values.

References

(1) Frank, T.; Shmueli, S.; Cohen Jungerman, M.; Shekhter, P.; Selzer, Y. Large Seebeck Values in Metal–Molecule–Semimetal Junctions Attained by a Gateless Level-Alignment Method. *Nano Lett.* **2023**, *23* (22), 10473–10479.

(2) Shmueli, S.; Cohen Jungerman, M.; Shekhter, P.; Selzer, Y. Efficient Molecular Rectification in Metal–Molecules–Semimetal Junctions. *J. Phys. Chem. Lett.* **2024**, *15* (42), 10602–10608.

(3) Cohen Jungerman, M.; Shmueli, S.; Shekhter, P.; Selzer, Y. Unusually High Thermopower in Molecular Junctions from Molecularly Induced Quantized States in Their Semimetal Leads. *Nano Lett.* **2025**, *25* (7), 2756–2762.

(4) Lee, S.; Puck, A.; Graupe, M.; Colorado, R.; Shon, Y.-S.; Lee, T. R.; Perry, S. S. Structure, Wettability, and Frictional Properties of Phenyl-Terminated Self-Assembled Monolayers on Gold. *Langmuir* **2001**, *17* (23), 7364–7370.

(5) Sabatani, E.; Cohen-Boulakia, J.; Bruening, M.; Rubinstein, I. Thioaromatic Monolayers on Gold: A New Family of Self-Assembling Monolayers. *Langmuir* **1993**, *9* (11), 2974–2981.

(6) Tao, Y.-T.; Wu, C.-C.; Eu, J.-Y.; Lin, W.-L.; Wu, K.-C.; Chen, C. Structure Evolution of Aromatic-Derivatized Thiol Monolayers on Evaporated Gold. *Langmuir* **1997**, *13* (15), 4018–4023.

(7) Yang, Y.; Ruths, M. Friction of Polyaromatic Thiol Monolayers in Adhesive and Nonadhesive

Contacts. *Langmuir* **2009**, *25* (20), 12151–12159.

(8) Park, S.; Kang, S.; Yoon, H. J. Thermopower of Molecular Junction in Harsh Thermal Environments. *Nano Lett.* **2022**, *22* (10), 3953–3960.
